# Model Quality Assessment for CASP16

**DOI:** 10.1002/prot.70037

**Published:** 2025-08-22

**Authors:** Alisia Fadini, Gabriel Studer, Randy J. Read

**Affiliations:** ^1^ Cambridge Institute for Medical Research University of Cambridge Cambridge UK; ^2^ Biozentrum University of Basel Basel Switzerland; ^3^ SIB Swiss Institute of Bioinformatics, Computational Structural Biology Basel Switzerland

**Keywords:** computational molecular biology, molecular models, protein conformation, protein domains

## Abstract

The CASP16 evaluation of model accuracy (EMA) experiment assessed the ability of predictors to estimate the accuracy of predicted models, with a particular emphasis on multimeric assemblies. Expanding on the CASP15 framework, CASP16 introduced a new evaluation mode (QMODE3) focused on selecting high‐quality models from large‐scale AlphaFold2‐derived model pools generated by MassiveFold. Three primary evaluation tasks were therefore conducted: QMODE1 assessed global structure accuracy, QMODE2 focused on the accuracy of interface residues, and QMODE3 tested model selection performance. Predictors were evaluated using a diverse set of OpenStructure‐based metrics, and a novel penalty‐based ranking scheme was developed for QMODE3 to handle score interdependence and varying prediction quality distributions. Additionally, we explored the accuracy and utility of predicted local confidence measures now made available on a per‐atom basis by methods that invoke AlphaFold3. Results showed that methods incorporating AlphaFold3‐derived features—particularly per‐atom pLDDT—performed best in estimating local accuracy and in utility for experimental structure solution. For QMODE3, performance varied significantly across monomeric, homomeric, and heteromeric target categories and underscored the ongoing challenge of evaluating complex assemblies.

## 1 Introduction

The CASP experiments have traditionally been focused on assessing the quality of predicted atomic models by comparing the predictions to what was found by experimental structure determination. Since success in structure prediction requires one to distinguish high‐quality from poor‐quality structures, accuracy assessment has always implicitly measured accuracy self‐assessment, where confidence in model quality can be predicted before the answer is known. For a variety of reasons, model quality assessment has also developed as a separate category. First, it's an essential ingredient in meta‐predictors where models produced by a variety of methods are ranked. Second, explicitly testing accuracy self‐assessment can guide improvements in prediction methods. Third (and of great importance), models with confidence measures are far more useful to downstream users of those models.

### 1.1 Assessing Accuracy Self‐Assessment

Over the years, a large collection of model assessment criteria has accumulated to score both local and global features of models, so it is natural for predictors to attempt to predict how their models will score on these criteria. For a number of years, predictors were asked to predict the local root‐mean‐square (RMS) error of the atomic coordinates that would be found when the models were compared to experimental structures, and to place that estimate in the B‐factor column. However, a problem with the choice of the RMS error became more prominent in the assessment of template‐based modeling in CASP13, when RMS error estimates were used in evaluating the utility of models for molecular replacement [1]. The RMS error depends on how the model is aligned with the experimental structure, and it is poorly defined when the evaluation is carried out on relatively rigid evaluation units that are defined post hoc by quality assessors, and not on the full target. A measure that depends on local rather than global superposition is less dependent on evaluation unit definitions. Accordingly, the developers of AlphaFold2 [2] chose instead to predict the LDDT (local distance difference test [3]) score. The predicted LDDT (pLDDT) was adopted as the CASP community standard starting with CASP15.

In practice, the pLDDT has emerged as one of the most useful criteria for users of computational models. Thresholds of pLDDT values are used, for instance, in coloring AlphaFold2 models on the AlphaFold Database at the European Bioinformatics Institute (https://alphafold.ebi.ac.uk) [4]. In addition, when using computed models to solve experimental structures, the pLDDT values can be translated into crystallographic B‐factors to weight different parts of the model by their confidence, as well as to prune the least reliable segments [5].

Here we evaluate the pLDDT, both in its accuracy and in its utility for locally weighting models in experimental structure determination. One development since CASP15 is that pLDDT is now sometimes predicted per atom rather than per residue, so we also evaluate whether the finer‐grained prediction adds value.

### 1.2 Quality Assessment of Other Predictors' Models

#### 1.2.1 QMODE1/2: Global and Interface Quality Estimates

CASP15 witnessed a growing focus on assembly modeling, which was also reflected in the CASP15 evaluation of model accuracy (EMA) category [6]. Participants in the CASP15 EMA category were tasked with providing global quality estimates (QMODE1) for models generated in the assembly modeling category. Specifically, they were asked to provide two distinct estimates: one assessing the overall structural topology based on global superposition (*SCORE*), and another evaluating the accuracy of the predicted interfaces (*QSCORE*). In addition to global estimates, participants were also asked to provide local estimates for model interface residues (*Q*MODE2). These estimates aimed to determine whether a given residue is a correct interface residue also in the target structure, and the overall accuracy of the local environment, including neighboring chains (*Local*). This experiment was repeated in CASP16 with the goal of assessing progress and identifying areas for further improvement. A key limitation in CASP15 was, although many assembly predictors incorporated AlphaFold2 in some form [7], AlphaFold2‐derived global confidence scores such as interface pTM (ipTM) [8] were not consistently available to the assessors. Even though these scores are known to perform well [9], a consistent EMA baseline leveraging these scores was not established and directly compared to the performance of other EMA predictors. QMODE3 not only addresses this gap but also presents a real‐world challenge by requiring the selection of the best model from a complete set of predictions.

#### 1.2.2 QMODE 3: Ranking of MassiveFold Models

The Wallner group's strategy to massively sample AlphaFold2 proved to be very successful in CASP15 [10] and is now provided as a large‐scale model generation tool through MassiveFold [11]. For CASP16, the Brysbaert group generated 8040 MassiveFold models for each target (except smaller numbers for the largest ones) and made these available to the community [12]. For QMODE3 of the EMA experiment, accuracy estimators were asked to select the five best models from the MassiveFold model pool for each target.

**FIGURE 1:**
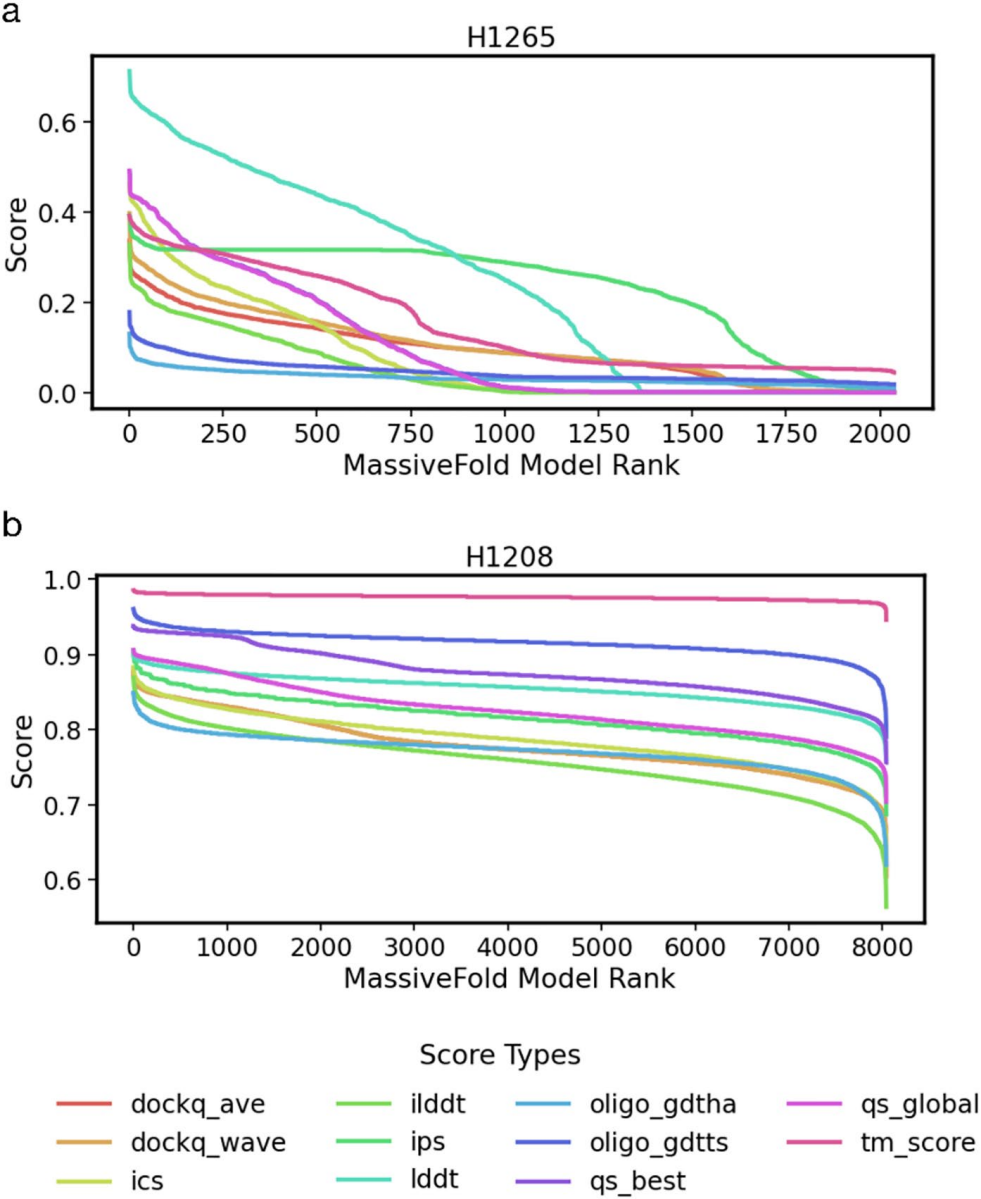
Quality distribution for MassiveFold models varies across targets. Each MassiveFold model for two heteromeric targets—H1265 (a) and H1208 (b)—is compared to the experimental structure through a specific OpenStructure (OST) score and all models are then ranked on the basis of that score. Score value is plotted against model rank for each of these comparisons.

**FIGURE 2:**
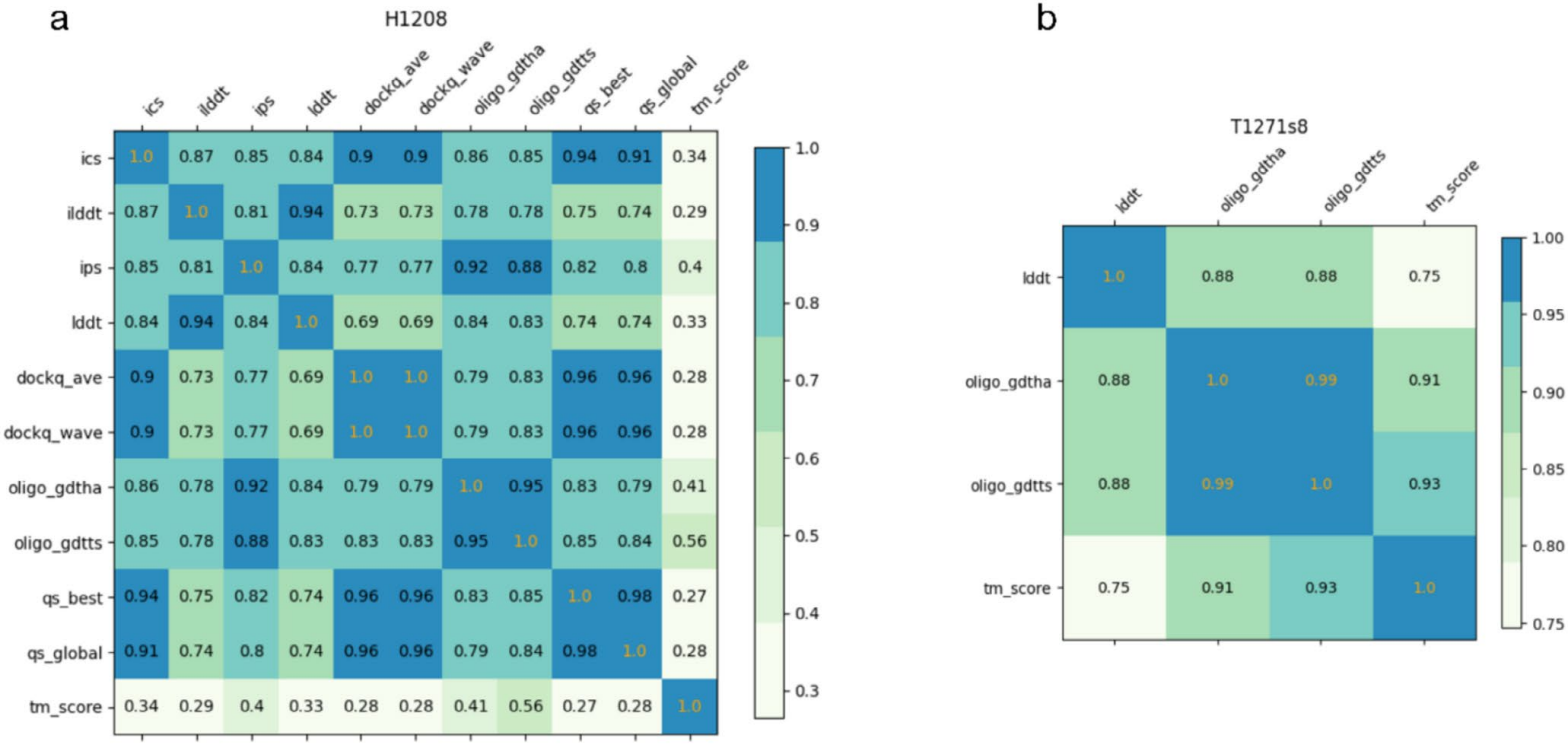
OpenStructure (OST) scores can be highly interdependent. Correlation matrices of OST scores obtained when comparing all MassiveFold models for a specific target to its respective experimental structure. An example for the set of OST scores used for heteromeric targets (H1208, a) and monomeric targets (subunit T1271s8, b) are shown.

Two important challenges arose in scoring QMODE3 predictor rankings. First, the distribution of MassiveFold prediction quality varied significantly across targets. For many targets, a large fraction of MassiveFold models were structurally similar, so a robust evaluation method had to account for this redundancy when it was present, as shown in Figure 1. As an extreme example, if the top 1000 models all had the same score for a particular criterion, it should not matter which 5 of those top 1000 models were selected to get a perfect score. Second, multiple structural assessment criteria exist to evaluate each MassiveFold prediction against an experimental target, requiring a single final score to combine these criteria in a meaningful way. We chose to use scores available in OpenStructure (OST) [13, 14, 15] to evaluate MassiveFold models against experimental target structures. Because such assessment criteria can be highly correlated (Figure 2), a sensible scoring scheme had to compensate for this to avoid over‐emphasizing particular model characteristics that were repeatedly tested.

Since predictors only provided their top five models rather than a complete ranking or confidence, standard score correlation metrics could not be applied directly. Instead, we developed a scoring approach that compares predictor selections against the full set of available models using a ranking error (RE)‐based penalty. To account for the relationships and correlations between different OST scores, we weighted their contribution to the final penalty through their covariance.

## 2 Methods

For most assessments, rankings were computed using the traditional CASP *Z* score calculation [3] for each ranking criterion: the mean and standard deviation of each criterion among all the models were computed for each target; then used to convert the ranking scores into *Z* scores. In the first pass of this algorithm, the mean and standard deviation were computed from all models for a target. In a second pass, groups with scores worse than two (or sometimes three) standard deviations below the mean were eliminated, and a revised mean and standard deviation were computed as the basis for the final *Z* score. All negative *Z* scores were then assigned a value of zero [3]. In most cases, as noted below, missing predictions were mildly penalized by assigning a *Z* score of zero.

### 2.1 Accuracy Self‐Assessment by pLDDT

The accuracy and utility of pLDDT estimates were assessed against predictions for 93 domain evaluation units from protein models. Statistics were collected for the 54 predictors who participated for at least 80% of these targets.

#### 2.1.1 pLDDT Accuracy

As noted above, users of predicted models interpret the actual pLDDT values, not just the relative values within a model. For that reason, as done in CASP15, we chose to assess pLDDT accuracy by the deviation between the predicted and actual values, rather than by (for instance) a correlation coefficient [6]. However, the CASP15 metric was based on the absolute value of the deviation, whereas we chose the root‐mean‐square deviation (RMSD) (in practice, this choice had a negligible effect on the results such as ranking order of different predictors). The LDDT values for the predicted models were computed using OST [14, 15] version 2.9, in which a new feature was added to enable per‐atom LDDT scores. The pLDDT RMSD scores were computed both per‐residue and per‐atom; for models provided with per‐atom pLDDT values, the per‐residue scores were calculated using the average pLDDT value for the atoms in a residue, whereas for models provided with only per‐residue pLDDT values, the per‐atom scores were calculated by assigning the same pLDDT value to each atom in a residue. All five predictions provided for each target were evaluated, on the basis that the accuracy of a model should be predicted well regardless of its actual accuracy. The ranking score was computed using the traditional CASP *Z*‐score algorithm, as discussed above. *Z*‐scores of zero were assigned to missing predictions.

#### 2.1.2 pLDDT Utility for Structure Solution

The placement of atomic models to explain either experimental crystallographic diffraction data or cryo‐EM maps can be evaluated by the log‐likelihood gain (LLG) that measures agreement with the experimental data. The actual LLG value obtained can be interpreted as a confidence level, with LLG values greater than about 60 typically being associated with correct placements either for molecular replacement in crystallography [16] or docking in cryo‐EM [17]. The utility of a model for solving an experimental structure can therefore be evaluated by the fraction it would give of the LLG score that would be obtained from an ideal model. This can be assessed by the relative expected LLG (reLLG) score introduced for CASP14 [18]. Although it would also be sensible to rank the utilities of the models themselves by the best model provided by each group, for the assessment of pLDDT utility we preferred to look at its impact for all five models provided for each target. The impact of the pLDDT estimate was measured using the gain in reLLG comparing the accuracy‐weighted model with an unweighted model. In addition, the significance of the individual atomic pLDDT estimates was measured using the gain in reLLG when the individual estimates were used instead of their per‐residue averages.

As done for pLDDT accuracy, the ranking score was computed using the traditional CASP Z‐score algorithm; missing predictions were assigned a Z‐score of zero.

### 2.2 QMODEs 1 and 2

We obtained EMA predictions for 13 640 models, 5 605 282 interface residues respectively, from 41 targets in the CASP assembly prediction category. The CASP organizers provided the expected stoichiometry for each target to the prediction groups in the assembly prediction category, and we excluded all models with stoichiometry differing from that of the target structure (approximately 3% of the models) from our analysis. This restricts the EMA prediction task to accuracy estimation under the assumption of correct stoichiometry. To ensure a robust comparison, we only evaluated EMA predictors that returned at least 80% of the expected data points for at least 80% of the 41 CASP targets. For example, in a target with 300 models totaling 130 000 interface residues, an EMA method must return at least 240 QMODE1 scores and 104 000 QMODE2 scores. This threshold must be met for at least 33 targets. Based on this criterion, the QMODE1 evaluation included 24 EMA predictors for *SCORE* and 25 for *QSCORE* (Figure S1). 15 EMA predictors fulfill the equivalent criterion for *Local* predictions in QMODE2 (Figure S2). Evaluation and ranking largely follow the methodologies established in CASP15 [6], and the following is a summary that highlights the key differences.

*SCORE* predictions of QMODE1 are assessed using Oligo‐GDTTS [6] computed by OST and TM‐score [19] computed by USalign [20], which are both global superposition‐based reference values that capture overall topology. In contrast, *QSCORE* predictions in QMODE1 are assessed using QS‐score [13] and DockQ‐wave [6] computed by OST, which specifically evaluate interface quality. As in CASP15, the QS‐best variant is used for QS‐score. It only evaluates the parts that are covered in both model and target. The reason for this is that the symmetry of the original QS‐score does not distinguish between model and target in order to establish a similarity measure between the two quaternary states. However, this gives a lower score if parts of the target structure are not resolved, essentially penalizing interfaces in the model for which no experimental data exist.

The assembly consensus baseline from CASP15 [6], here named “AC Baseline,” was used to create predictions for *SCORE* and *QSCORE*. This baseline score is the average of either *SCORE* or *QSCORE* for the tested model against all other models. Consensus methods historically achieved good results in the CASP context, but they are heavily dependent on the target and the diversity of the underlying models.

*Local* predictions in QMODE2 are evaluated based on their ability to classify model interface residues as true interface residues, those also present at the interface in the reference structure, or as non‐interface residues. The ground truth is determined by identifying whether a model interface residue corresponds to an interface residue in the target structure, based on a residue‐level mapping established using the OST chain mapping algorithm. Predictors are evaluated based on their ability to distinguish between these two classes using the average per‐target area under the curve (AUC) from a receiver operating characteristic (ROC) analysis. Beyond this task, we extend the evaluation to also consider a predictor's capacity to estimate the accuracy of model interface residues, accounting for their local structural environment and specific interactions with neighboring chains. Local accuracy is quantified using the per‐residue LDDT [21] CAD (AA variant) [22], PatchQS [6], and PatchDockQ [6] reference values as computed in OST. PatchQS and PatchDockQ were introduced in CASP15 as a complement to LDDT and CAD to better address inter‐chain aspects. LDDT and CAD capture pairwise interactions and contact areas, respectively, based on the reference structure and evaluate how well these features are reproduced in the model. While they are both useful measures to assess the local full atomic environment of a residue, they do not penalize incorrect contacts that appear exclusively in the model—an issue particularly relevant in a local context to identify inaccurately modeled interfaces. To better emphasize the inter‐chain aspects of LDDT in CASP16, we employed a variant of LDDT that also considers model‐specific contacts that would otherwise be excluded. Standard LDDT extracts a set of contacts from the reference structure using a pairwise distance threshold. The modified variant extends this approach by incorporating additional contacts from the model if they satisfy the threshold in the model alone, provided the corresponding residues can be mapped to the reference structure and are thus covered by experimental data.

Pearson (*P*) and Spearman (*S*) correlation coefficients, ROC AUC (*R*), and loss (*L*) metrics, as used in CASP15, are employed to assess the relationship between EMA predictions and reference values. Loss (*L*) is the difference between the reference value of the model identified as top‐ranked by a given predictor and the best reference value observed among all candidate models. In the example of Pearson correlation (*P*), the computation for a given reference value *r* and EMA predictor *p* is defined as

$$P\left(r,p\right)=\sum_{t}^{\text{Targets}}\max\left(0,\frac{P\left(r,p,t\right)-\mu \left(P\left(r,t\right)\right)}{\sigma \left(P\left(r,t\right)\right)}\right)$$

where $P\left(r,p,t\right)$ is the Pearson correlation for predictor *p*, when compared with reference value *r* on CASP prediction target *t*. $\mu \left(P\left(r,t\right)\right)$ and $\sigma \left(P\left(r,t\right)\right)$ are the mean and standard deviation, respectively, across all predictors. This approach sums per‐target *Z*‐scores to assess predictor performance relative to the average. Targets with no predictions are penalized by adding 0.0 to the overall sum. Notably, this differs from the methodology employed in CASP15, where *Z*‐scores were computed after averaging correlations across targets. For QMODE1, we define a ranking score (RS) for a given reference value *r* as

$${\mathrm{RS}}_{\text{QMODE}1}\left(r,p\right)=0.5\times P\left(r,p\right)+0.5\times S\left(r,p\right)+R\left(r,p\right)+L\left(r,p\right)$$

The final ranking scores in QMODE1 are computed as follows:

$${\mathrm{RS}}_{\text{SCORE}}\left(p\right)={\mathrm{RS}}_{\text{QMODE}1}\left(\text{Oligo}-\text{GDTTS},p\right)+{\mathrm{RS}}_{\text{QMODE}1}\left(\mathrm{TM}-\text{score},p\right)$$

$${\mathrm{RS}}_{\text{QSCORE}}\left(p\right)={\mathrm{RS}}_{\text{QMODE}1}\left(\mathrm{QS}-\text{score},p\right)+{\mathrm{RS}}_{\text{QMODE}1}\left(\text{DockQ}-\text{wave},p\right)$$

For QMODE2, the loss (*L*) is excluded, resulting in the ranking score:

$${\mathrm{RS}}_{\text{QMODE}2}\left(r,p\right)=0.5\times P\left(r,p\right)+0.5\times S\left(r,p\right)+R\left(r,p\right)$$

The final ranking score for QMODE2 is given by

$$\begin{array}{c} {\mathrm{RS}}_{\text{Local}}\left(p\right)={\mathrm{RS}}_{\text{QMODE}2}\left(\text{LDDT},p\right)+{\mathrm{RS}}_{\text{QMODE}2}\left(\mathrm{CAD},p\right)\\ \;+{\mathrm{RS}}_{\text{QMODE}2}\left(\text{PatchQS},p\right)+{\mathrm{RS}}_{\text{QMODE}2}\left(\text{PatchDockQ},p\right) \end{array}$$

For the evaluation of *SCORE*, only targets for which at least one model achieved a TM‐score ≥ 0.6 were included (38 targets). Similarly, the evaluation of *QSCORE* and *Local* was limited to targets with at least one model attaining a QS‐score ≥ 0.6 (39 targets).

### 2.3 QMODE 3

We divided targets into three categories: monomers, homomers, and heteromers. For each MassiveFold model, we used OST to compute a set of structural scores against its experimental target: LDDT, TM‐score, QS‐global, QS‐best, RMSD, oligo‐GDTHA, oligo‐GDDTS for all targets, and iLDDT, IPS, ICS, DockQ‐ave, and DockQ‐wave for oligomeric targets [13, 21]. (Note that oligo‐GDTHA and oligo‐GDTTS are equivalent to GDTHA and GDTTS in the case of monomers.) RMSD, IPS, and ICS values are available in our depositions but were not used for the following weighted penalty calculations. To participate in QMODE3, we included predictor groups that had submitted predictions for at least 80% of the relevant targets. There was no additional penalty if a predictor did not return predictions for certain targets as long as this requirement was met.

Each predictor's ranking of five models was compared against a true ranking derived from the full set of MassiveFold models based on a specific OST score. For each target, a RE was assigned for each OST score to quantify how far the predictor's ranking deviated from the true ranking. The RE was computed as

$${\mathrm{RE}}_{\mathrm{ij}}=\sum_{k=1}^{5}\left|S_{\text{true}}\left(k\right)-S_{\text{pred}}\left(k\right)\right|$$

where $S_{\text{true}}$ and $S_{\text{pred}}$ represent the OST score values for the *k*th model in the true and predicted lists, respectively. The RE could therefore range from zero (for perfect ranking) to a positive value that depended on the quality of MassiveFold models for that target, how badly the predictor ranked those models, as well as on the range of values for that OST score. For each predictor group, this resulted in a RE matrix of dimensions (number of OST scores by number of targets).

As mentioned above, many of the OST scores are highly interdependent, meaning that a naive summation of REs would overemphasize redundant features. To combine the REs from different OST scores into a final penalty, we used the Mahalanobis distance [23], which takes account of covariances to transform coordinates into a principal coordinate space, thus yielding a kind of *n*‐dimensional *Z*‐score. Mathematically, the weighted overall penalty for a predictor group was computed with a triple product:

$$P_{w}=\sqrt{\mathrm{RE}\sum^{-1}{\mathrm{RE}}^{T}}$$

where RE is the ranking error vector for a specific OST score and ∑ is the covariance matrix indexed by the different OST scores. The weighted penalty $P_{w}$ can, therefore, be thought of as the Mahalanobis distance from a perfect penalty score of all zeroes.

We computed the covariance matrix of OST scores from the variation among the entire set of models for all the targets in a particular category (i.e., monomer, homomer, heteromer), rather than from just the models chosen by one or more of the predictors, in order to capture the full behavior of the criteria. We constructed separate covariance matrices for the different categories of targets because different OST scores are available for multimers than for monomers, and behavior differs between homomers and heteromers. We also experimented with a separate covariance matrix per target, but this made little difference to the end results while being harder to justify in terms of changing the relative weights of criteria among targets. An additional complication in the computation of the covariance matrices arose from the presence of structural outliers—particularly in multimeric targets—where some models displayed severe steric clashes or were evidently mispredicted. For this reason, we computed covariance matrices in two passes: the first pass covered all models for all relevant targets. The Mahalanobis distance from the center of the distribution was then used to screen out severe outliers before computing the final covariance matrix on a second pass.

Finally, the weighted penalty scores were normalized on a per‐target basis to compare predictors equally across targets of varying difficulty. This normalization step rescales penalties between 0 and 1, using

$$P_{w_{\mathrm{norm}}}=\frac{P-P_{\min}}{P_{\max}-P_{\min}}$$

preventing the final scoring from being dominated by particularly challenging targets while still reflecting how well a specific predictor did, compared to other predictors, with the best predictor getting a penalty of zero and the worst predictor getting a penalty of one.

As an additional analysis, we used a secondary method to process REs across targets and OST scores. This was a traditional CASP *Z*‐score calculation [24] for each criterion, differing only in using a threshold of three standard deviations below the mean to filter out results from the worst‐performing groups. In contrast to the weighted penalty calculation, we used equal weights for all OST criteria to combine *Z*‐scores into final rankings. We used this equally weighted *Z*‐scores approach as a way of cross‐checking our Mahalanobis‐inspired approach, and found that the two methods yielded similar results for the final rankings.

As baselines across QMODE3, we used MassiveFold's internal confidence scores: pLDDT and pTM for monomers and, for oligomers, the additional ipTM and ipTM + pTM metrics.

## 3 Results

### 3.1 Accuracy Self‐Assessment by pLDDT

We evaluated the results from 93 domain evaluation units derived from protein targets. Fifty‐four predictors participated for at least 80% of targets. The majority of these predictors provided only per‐residue pLDDT values, but 25 predictors provided finer‐grained per‐atom values for at least half of their models.

#### 3.1.1 pLDDT Accuracy

**FIGURE 3:**
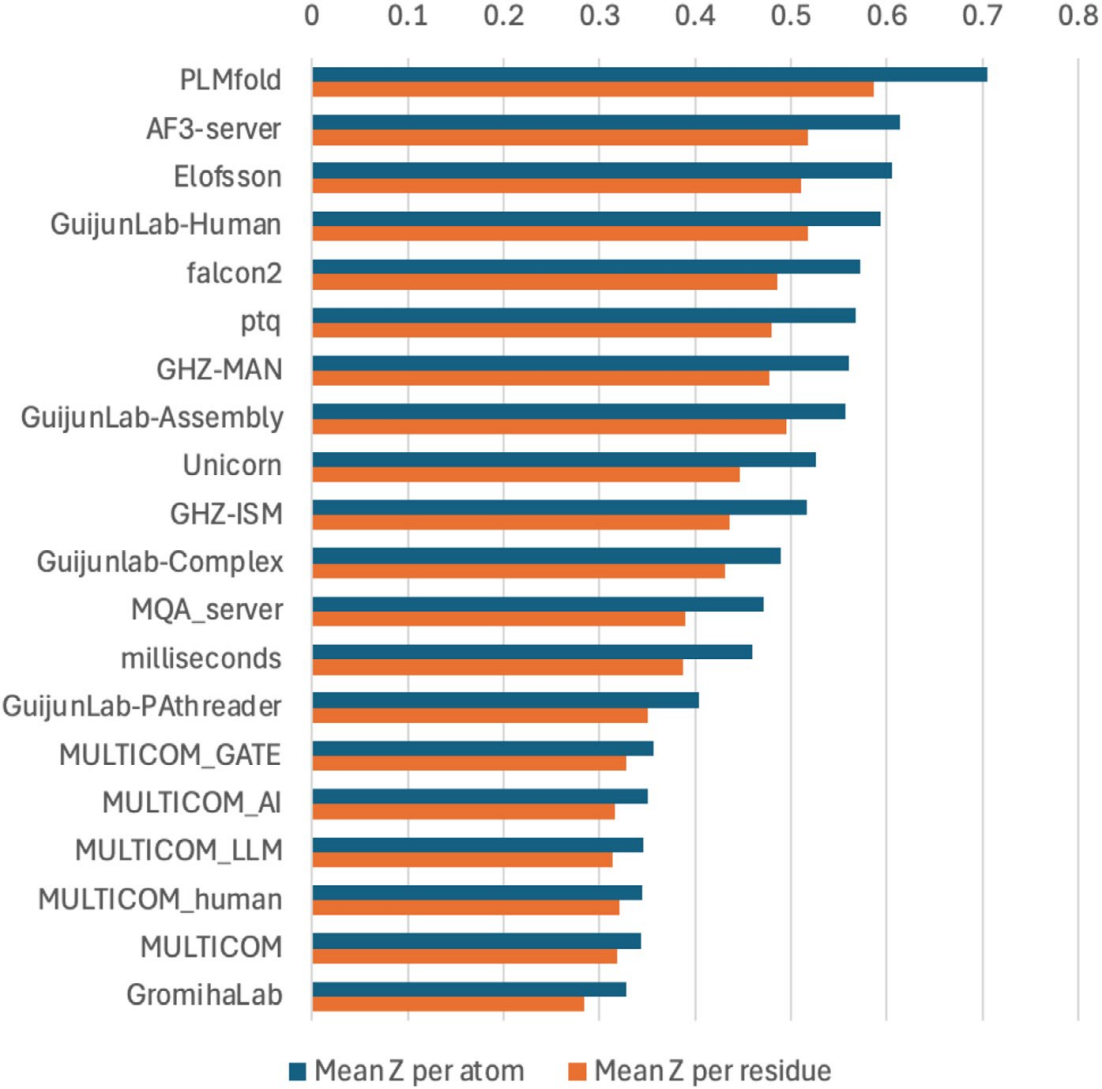
*Z*‐score ranking of pLDDT RMSD for the top 20 groups, on both a per‐atom and a per‐residue basis.

Initially, we observed substantial differences among groups in the accuracy of pLDDT estimates. However, as we attempted to discover what the best predictors had done well, it emerged that all of the best results came from the 25 predictors who provided primarily per‐atom pLDDT estimates. This was true regardless of whether the pLDDT RMSD values were evaluated on a per‐atom or per‐residue basis, as shown in Figure 3. Communication with the top predictors revealed that all of them had used AlphaFold3 [25] as part of their pipelines. There was also a strong trend for the quality of pLDDT estimates to correlate with the fraction of models that contained individual pLDDT estimates (and therefore would have been derived from AlphaFold3 models). Indeed, the second‐best results (judged by per‐residue RMSD) came from the AF3‐Server group [26], which used AlphaFold3 for all submitted models. As a result, our main conclusion is that AlphaFold3 has improved methods for pLDDT estimation, compared to AlphaFold2. Part of this improvement, but not all, comes from a better atom‐by‐atom agreement with the per‐atom LDDT values.

An interesting exception was the PLMfold group, which would seem to have enhanced the accuracy of the pLDDT estimates that were originally obtained from AlphaFold3. However, discussions with the group members did not reveal features of their algorithms that would result in such improvements (Wei Liu, personal communication). In addition, a close examination of the per‐target results suggests that the apparent improvement over AF3‐Server could largely be random, as it disappears after removing only the top 3 of the 93 targets.

#### 3.1.2 pLDDT Utility

As we have shown before [5, 18, 27], weighting models by local confidence improves signal in molecular replacement or docking searches, thereby adding utility. On average, comparing the reLLG scores for unweighted models with those weighted according to the pLDDT, the weighting increases the score by about 15%. A significant part of this increase can be attributed to the fine‐grained pLDDT values. Taking the results from AF3‐Server as an example, the average reLLG score for unweighted models was 13.0. This increased to 16.3 when the models were weighted using coarse‐grained per‐residue pLDDT values, and further to 17.6 using the fine‐grained pLDDT estimates.

### 3.2 QMODEs 1 and 2

**FIGURE 4:**
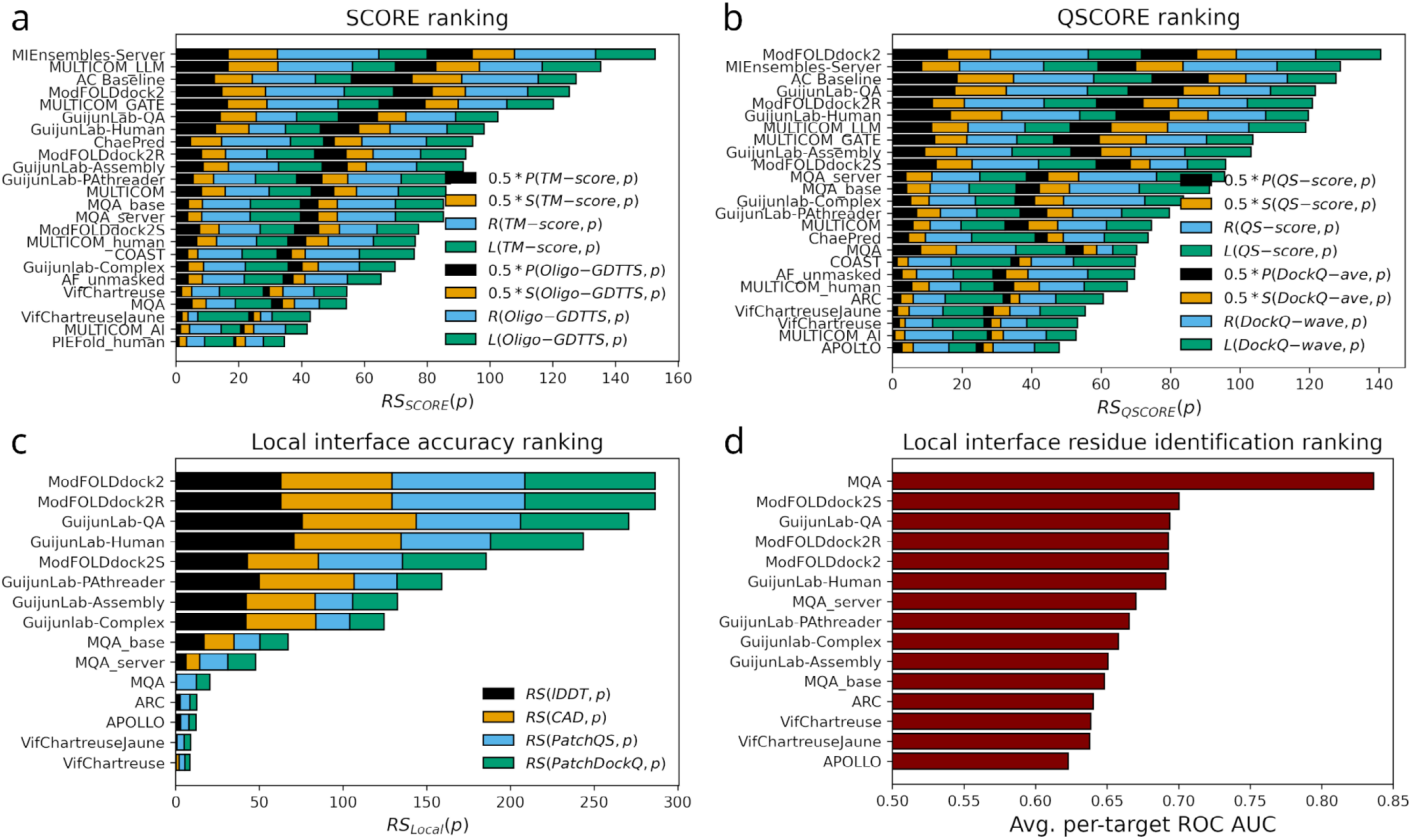
Rankings for QMODE1/2. (a) Overall *Z*‐score based *SCORE* ranking using superposition dependent reference values TM‐score and oligo‐GDTTS. (b) Overall *Z*‐score based *QSCORE* ranking using interface centric reference values QS‐score and DockQ‐wave. (c) *Local* interface accuracy ranking based on *Z*‐score using LDDT, CAD PatchQS, and PatchDockQ as reference values. (d) *Local* interface residue identification ranking assessing a predictor's capacity to identify model interface residues that are true interface residues, that is, part of an interface in the native structure. For (a and b), *P* is the Pearson correlation coefficient, *S* is the Spearman correlation coefficient, *R* is the area under the ROC curve, and *L* is the Loss function.

The AC Baseline remains a strong benchmark for EMA methods in the QMODE1 setting and, as in CASP15, it consistently ranks near the top (Figure 4a,b). Only a few methods outperform it in either the SCORE or QSCORE rankings, and they use consensus‐based information to varying degrees. Among them, ModFOLDdock2 and MULTICOM_LLM closely follow a traditional consensus‐based strategy by conducting all‐vs‐all comparisons in the input model set. ModFOLDdock2 additionally integrates contributions from the single‐model method VoroIF [28] in its QSCORE predictions. MIEnsembles‐Server, besides leveraging methods assessing individual models, also integrates consensus information but generates its own set of models using high quality multiple sequence alignments (MSAs) from DMFold [29] for the comparisons, rendering it independent of the original input model set. Although it functions as a single‐model method in practice, it is better described as a quasi‐single‐model approach.

In terms of absolute performance, Local interface accuracy assessment has clearly improved since CASP15 across all reference values (Table 1). The top four methods in the Local ranking (Figure 4c), ModFOLDdock2, ModFOLDdock2R, GuijunLab‐QA, and GuijunLab‐Human, are consensus methods that depend on the input model set. Notably, ModFOLDdock2S, ranked fifth, represents a quasi‐single‐model method that is complemented by contributions from single model methods, while GuijunLab‐PAthreader, ranked sixth, is the highest‐performing true single‐model method. Along with MIEnsembles‐Server, which participated in QMODE1, these two methods are particularly valuable as they are suited to assess individual models without requiring access to a large ensemble of alternatives.

**TABLE 1:** Average per‐target Pearson correlations ± SD (proxy for absolute performance) for top‐three groups on *Local* data in CASP15 and groups ranked 1, 3, and 4 in CASP16 (the two top‐ranked groups in CASP16 returned the exact same predictions). LDDT w/o added model contacts (computed as in CASP15) shown in brackets for CASP16.

	LDDT	CAD	PatchDockQ	PatchQS
CASP15	0.50 ± 0.17	0.44 ± 0.16	0.41 ± 0.22	0.41 ± 0.22
CASP16	0.72 ± 0.17 (0.67 ± 0.17)	0.61 ± 0.15	0.76 ± 0.28	0.75 ± 0.28

Using the same EMA predictions to classify model interface residues as true interface residues, that is, those also present in the native interface, highlights a clear top performer: MQA (Figure 4d). Notably, the two *Local* tasks are inherently different. A residue may be correctly identified as part of an interface while its inter‐chain interactions are inaccurately modeled. To the best of the assessors' knowledge, MQA was the only method to explicitly address the challenge of identifying true interface residues by estimating the probability of interface participation using the full model ensemble as input.

**FIGURE 5:**
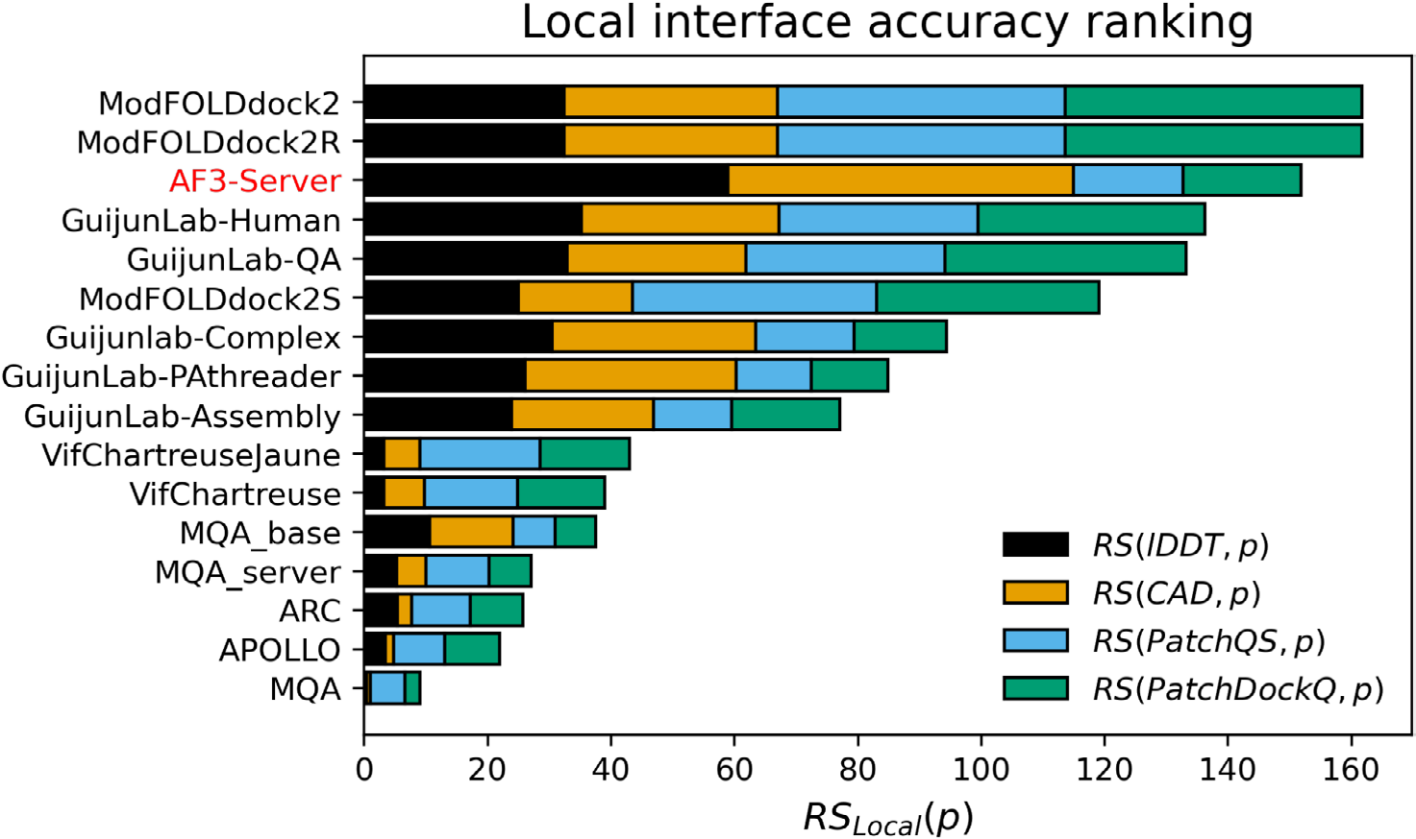
Local interface accuracy analysis on models from AF3‐Server. Analysis on 130 models across 26 targets (targets filtered for at least one model with QS‐score ≥ 0.6). AF3‐server (red) included as EMA predictor using per‐residue B‐factor averages as predictions.

In a final experiment, we compared *Local* predictions from EMA methods with self‐confidence estimates (Figure 5). The comparison is inherently imbalanced. A modeling method has full knowledge of its own model construction process, whereas EMA methods operate independently but, in the case of consensus strategies, can rely on a diverse set of models available in the CASP context. The *Local* accuracy assessment pipeline was applied to 130 models across 26 targets generated by AF3‐Server, selected based on the criterion that at least one model per target achieved a QS‐score ≥ 0.6. AF3‐Server, which showed strong performance in the self‐confidence evaluation (Figure 3), was included as an additional EMA method by using average per‐residue pLDDT values as confidence predictions. Notably, for interface residues, AF3‐Server ranked among the top EMA methods. Its performance was particularly strong for LDDT and CAD, while it was less accurate for inter‐chain specific metrics such as PatchDockQ and PatchQS. This is expected, as AF3‐Server's self‐confidence scores (pLDDT) are specifically optimized to reflect LDDT‐like measures. Similar trends were observed for other top‐five methods in Figure 3 (data not shown), suggesting that the self‐confidence estimates in CASP16 are on par with *Local* predictions from dedicated EMA methods.

### 3.3 QMODE 3

**FIGURE 6:**
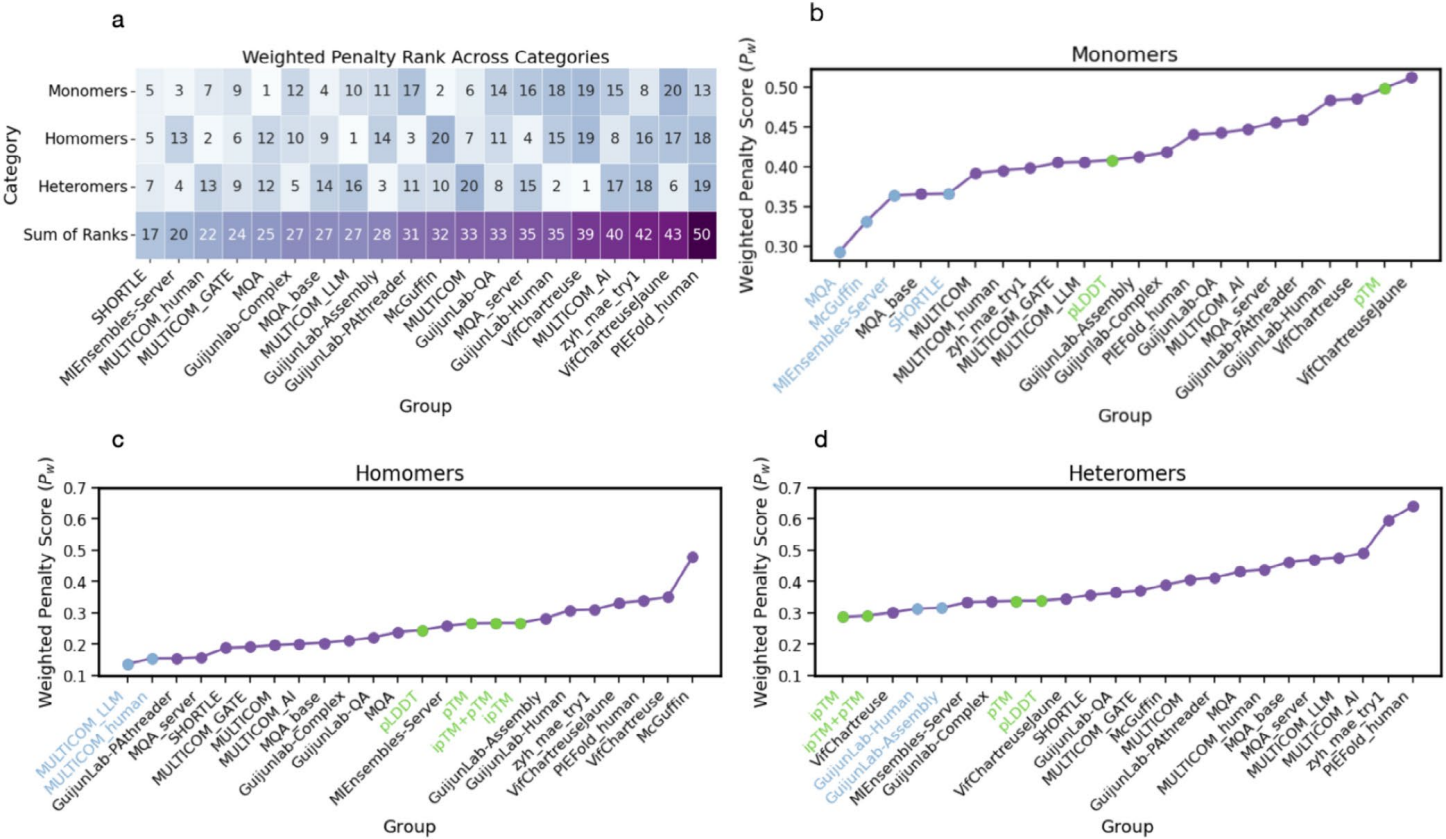
QMODE3 weighted penalty group rankings per target category. (a) The final rank obtained by each predictor group through the QMODE3 covariance‐weighted penalty is shown for three different target categories. The self‐confidence baselines from MassiveFold are not included for this plot. The sum of ranks obtained across each category is also shown for each group. (b–d) QMODE3 weighted penalty values obtained from each category are plotted against ranked predictor group. Groups that were robustly in the top five positions for variations of the analysis (including a traditional *Z*‐score ranking that equally weighted score contributions—see Section 2) are highlighted in light blue. Baseline methods (i.e., evaluation of MassiveFold models predicted solely through its internal confidence metrics) are highlighted in green.

Figure 6 shows final weighted penalty rank results for predictor groups in the three target categories. A first observation is that group performance varied significantly across categories. A sum of the three ranks provides an indication of which methods proved the most general across categories and highlights the SHORTLE and MIEnsembles‐Server in the top two positions. When analyzing weighted penalty values per group for the separate categories (Figure 6b–d), it is apparent that the rankings are not very steep, with the top ten groups performing very similarly to each other, particularly in the assessment of oligomers. This means that the exact rankings can be susceptible to small differences in the analysis. For this reason, we chose to highlight groups (light blue) that were consistently in the top five positions when different methods were used to produce the final rankings (i.e., the per‐category score covariance‐weighted penalty shown in Figure 6a, a per‐target score covariance‐weighted penalty, and the *Z*‐score based assessment described in Section 2).

Interestingly, two of the groups that stand out for the monomer category—SHORTLE and MIEnsembles‐Server—were already mentioned for their generality across categories. These two well‐performing strategies are orthogonal. MIEnsembles‐Server, briefly described in the previous section, relies extensively on models generated by the AlphaFold2‐based DMFold pipeline and deep learning‐based predictors that assess individual models. On the other hand, SHORTLE did not rely on a deep learning approach but on a collection of statistical parameters and potentials to score predictions. These scores were then compared with the scores of a set of 6000 high resolution structures from the PDB [30] to judge quality. We investigated the possibility that our chosen set of OST scores would skew the sum of ranks across categories towards methods that are successful in monomer prediction; we therefore recomputed weighted penalties for the homomer and heteromer categories using only DockQ_ave, DockQ_wave, and iLDDT metrics (Figure S3). This analysis still promotes the SHORTLE and MIEnsembles‐Server groups to the top two positions when summing final ranks across the three target categories. Interestingly, the SHORTLE group is top performing even when the monomer category is excluded entirely (Figure S3a), despite its high resolution reference set being a collection of only single chain structures. The McGuffin and MQA groups, in the top two positions for the QMODE3 monomer category, were also prominent in QMODE1 and QMODE2 outlined above.

For the homomer category (Figure 6c), MULTICOM_LLM and MULTICOM_human are robustly in the top two positions for the final ranking. For both monomers and homomers, the majority of groups outperform baseline AlphaFold2 confidence metrics; this is, however, not the case for heteromers (Figure 6d), where ipTM and pTM outperform predictors in weighted penalty. GuijunLab‐Human and GuijunLab‐Assembly are found consistently in the top five for heteromers. A comparison of Figure 6c,d makes it clear that there is still much room for improvement in ranking heteromer models.

Previous analyses of model selection from MassiveFold datasets [11] highlighted that more accurate models are often present in large sampling datasets but not selected by scoring strategies. To investigate this aspect in the CASP16 QMODE3 task, we examined, for each target, which model (from any group) achieved the lowest RE. We then asked where that model actually ranked in the true model list based on DockQ_ave (for oligomers) or TM score (chosen as a less DockQ‐correlated global accuracy metric that is applicable to all target categories). On average, the best‐selected model had a true rank of 147 (TM‐score) or 62 (DockQ_ave), possibly confirming previous observations that current EMA methods still struggle to identify the top‐quality models available in the prediction model pools. We note, however, that the very worst cases involved a target with many structurally similar models (for T1292 many models had near‐identical TM‐scores), or a generally poor overall model pool to pick from (DockQ_ave scores were below 0.3 for all predictions for T1249v2). These examples underline the importance of accounting for dataset distributions in the RE scheme and suggest that the mean true ranks of the best‐selected models underestimate true performance. Further, the true top models were correctly chosen by at least one predictor group for 10 targets each in both the TM score and DockQ_ave analyses (two examples are shown in Figure S4), which is in itself a promising result for the EMA category.

## 4 Conclusions

EMA continues to be an essential aspect of structure prediction, both in guiding the algorithms of the best methods and in guiding others to make the best possible use of structural models. Self‐assessment by the modeler of the accuracy of their own models should always play a key role, but there is still an important place for independent accuracy assessment. This is particularly relevant for areas where the results do not yet approach experimental accuracy, such as modeling complexes with other proteins or ligands or predicting alternative conformations.

In CASP16, as observed in CASP15 [6], most of the best‐performing methods are based on derivatives of AlphaFold2 [2] or AlphaFold3 [25]. We found little evidence that predictors altered the local (pLDDT) or global (pTM, ipTM) confidence estimates with their own algorithms, which makes comparisons among predictor groups less informative. The primary conclusion from the accuracy self‐assessment category looking at the accuracy of pLDDT was that estimates from AlphaFold3 seem to be more accurate than those from AlphaFold2 and that some of that improvement comes from giving finer‐grained per‐atom (rather than per‐residue) estimates. This improvement in accuracy gave a measurable improvement in the utility of the models for solving experimental structures by molecular replacement (crystallography) or docking (cryoEM).

The CASP16 EMA experiment revealed some clear advances in the assessment of interface quality (QMODE2), where EMA predictors demonstrated substantial improvements compared to CASP15. In QMODE3, which challenged predictors to select the top five high‐quality models from MassiveFold‐generated pools, group performance varied considerably between categories, suggesting a degree of specialization among methods. For heteromeric targets in particular, AlphaFold2 baseline confidence metrics (ipTM and ipTM + pTM) remained very competitive, indicating ongoing challenges for external assessment of complex assemblies. Interestingly, the SHORTLE group, whose method did not rely on deep learning, emerged as the most consistent performer across monomers, homomers, and heteromers.

Looking ahead to CASP17, we recommend that the organizers consider a number of refinements to the EMA categories. Improvements to pLDDT prediction could be encouraged by restricting the evaluation of local accuracy assessment to groups that do more than pass through the pLDDT values computed by algorithms from others, including AlphaFold2 and AlphaFold3. For QMODE1 and QMODE2, the analysis of the results would benefit from asking predictors for a clear indication of whether they are using single‐model or quasi‐single‐model methods (which are more generally relevant to downstream users) or consensus methods. Alternatively, we recommend an automated classification approach in which only a subset of models is initially submitted for each target, followed by submission of the full model set in a second stage. This two‐stage strategy, previously employed prior to CASP15, allows detection of consensus‐based scoring, as indicated by differences in predicted scores for models from the initial subset. For QMODE2 we recommend adapting the definition of the prediction task. Currently, predictors are asked whether a modeled interface residue corresponds to a true interface residue, regardless of the structural accuracy of the interface (evaluated in Figure 4d). However, the actual local accuracy of interface residues, as evaluated in Figure 4c, is more relevant to most end users and aligns more closely with the objective most predictors aim to address. Finally, we are pleased with the introduction of the QMODE3 task, both because larger‐scale sampling methods are becoming more common and because it avoids unnecessary duplication of computing effort. However, we suggest that it should be refined to require full model rankings rather than top‐five selections. This would enable better accounting for different model quality distributions across targets.

## Author Contributions

**Alisia Fadini:** conceptualization, investigation, writing – original draft, writing – review and editing, software, formal analysis. **Gabriel Studer:** conceptualization, investigation, writing – original draft, writing – review and editing, software, formal analysis. **Randy J. Read:** conceptualization, investigation, funding acquisition, writing – original draft, writing – review and editing, software, formal analysis, project administration, supervision.

## Conflicts of Interest

The authors declare no conflicts of interest.

## Supporting information


**Data S1:** Supporting Information.

## Data Availability

Assessment data are available from the CASP Prediction Center website, at https://predictioncenter.org/casp16. Code to reproduce the QMODE1/QMODE2 analysis is available at https://git.scicore.unibas.ch/schwede/casp16_ema. The analysis code for self‐assessment by pLDDT and for QMODE3 is available at https://github.com/alisiafadini/casp16.
