# Supplementary material for: Model Quality Assessment for CASP16
Source: Proteins. 2025 Aug 22;94(1):302–13. doi: 10.1002/prot.70037 (PMC12750031; doi:10.1002/prot.70037)
Supplement: Supplementary file 1 — Data S1: Supporting Information. [file PROT-94-302-s001.docx]

Supporting Information
Model Quality Assessment for CASP16

Running Title: Model Quality Assessment for CASP16

Authors: Alisia Fadini^1^, Gabriel Studer^2,3^ and Randy J. Read^1*^

^1^ Cambridge Institute for Medical Research, University of Cambridge, Cambridge, U.K.

^2^ Biozentrum, University of Basel, Basel, Switzerland

^3^ SIB Swiss Institute of Bioinformatics, Computational Structural Biology, Basel, Switzerland

^*^ Corresponding author

E-mail and ORCID:

Alisia Fadini, [af840@cam.ac.uk](mailto:af840@cam.ac.uk), [orcid.org/0000-0001-5246-9124](https://orcid.org/0000-0001-5246-9124)

Gabriel Studer, [gabriel.studer@unibas.ch](mailto:gabriel.studer@unibas.ch), [orcid.org/0000-0003-0462-1456](https://orcid.org/0000-0003-0462-1456)

Randy J. Read, [rjr27@cam.ac.uk](mailto:rjr27@cam.ac.uk), [orcid.org/0000-0001-8273-0047](https://orcid.org/0000-0001-8273-0047)


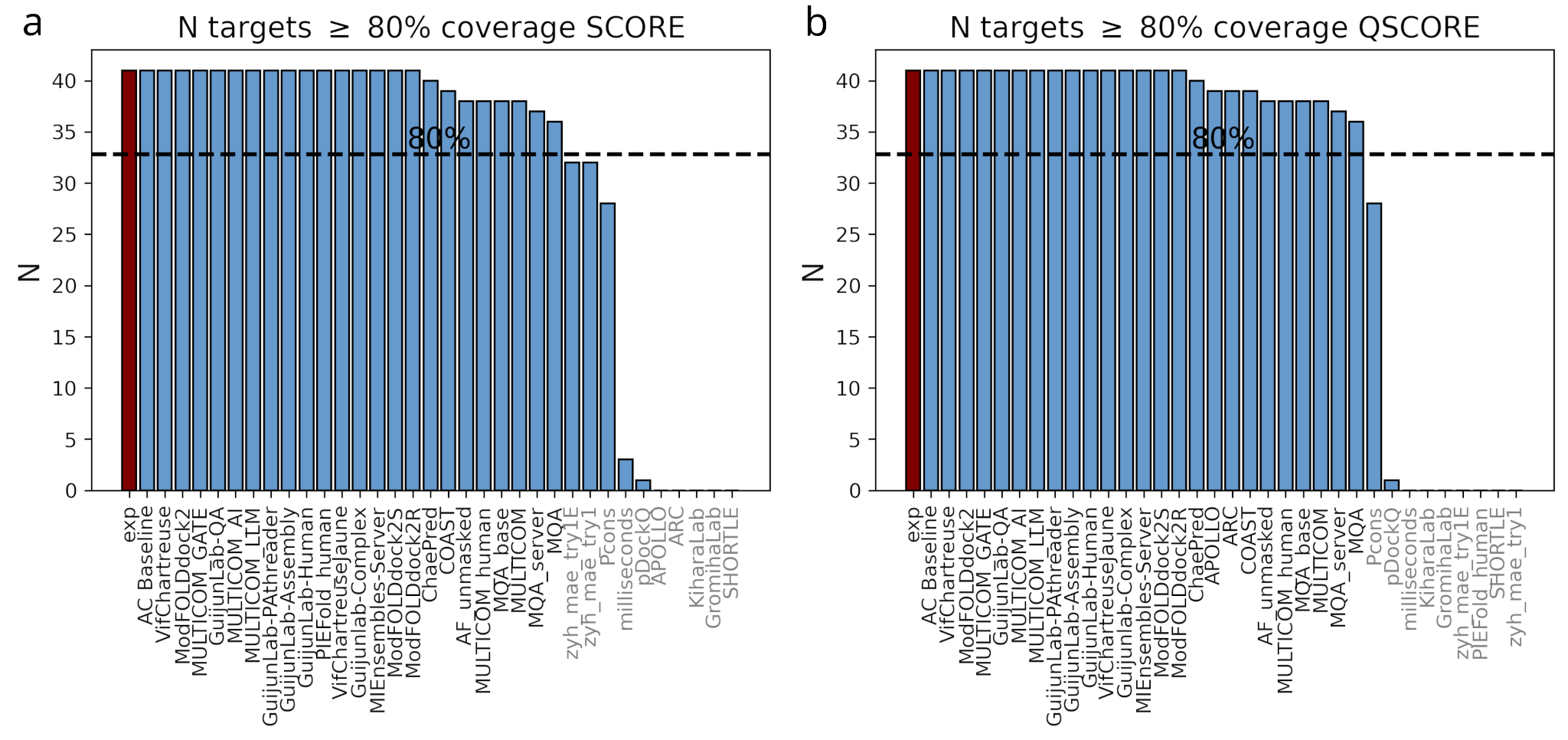


**Figure S1: QMODE1 data collection.** (a) EMA groups that returned 80% of expected SCORE data points for at least 80% of the targets (dashed line) are considered for evaluation. (b) QSCORE equivalent. “exp” represents the total number of evaluated targets and “AC Baseline” is the assembly consensus baseline. Grayed out methods do not fulfill the 80% threshold and are not evaluated.


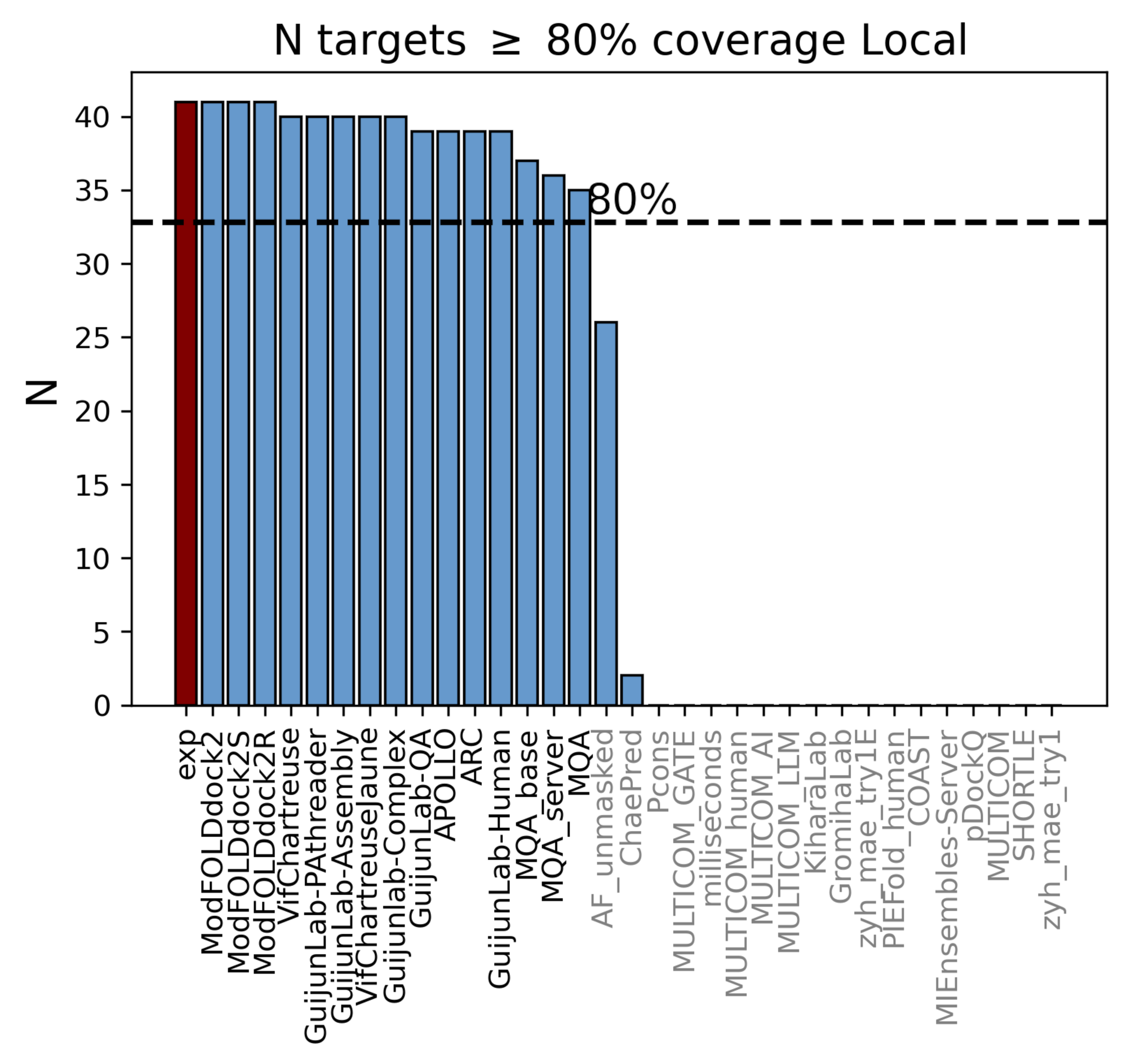


**Figure S2: QMODE2 data collection.** EMA groups that returned 80% of expected Local data points for at least 80% of the targets (dashed line) are considered for evaluation. “exp” represents the total number of evaluated targets. Grayed out methods do not fulfill the 80% threshold and are not evaluated.


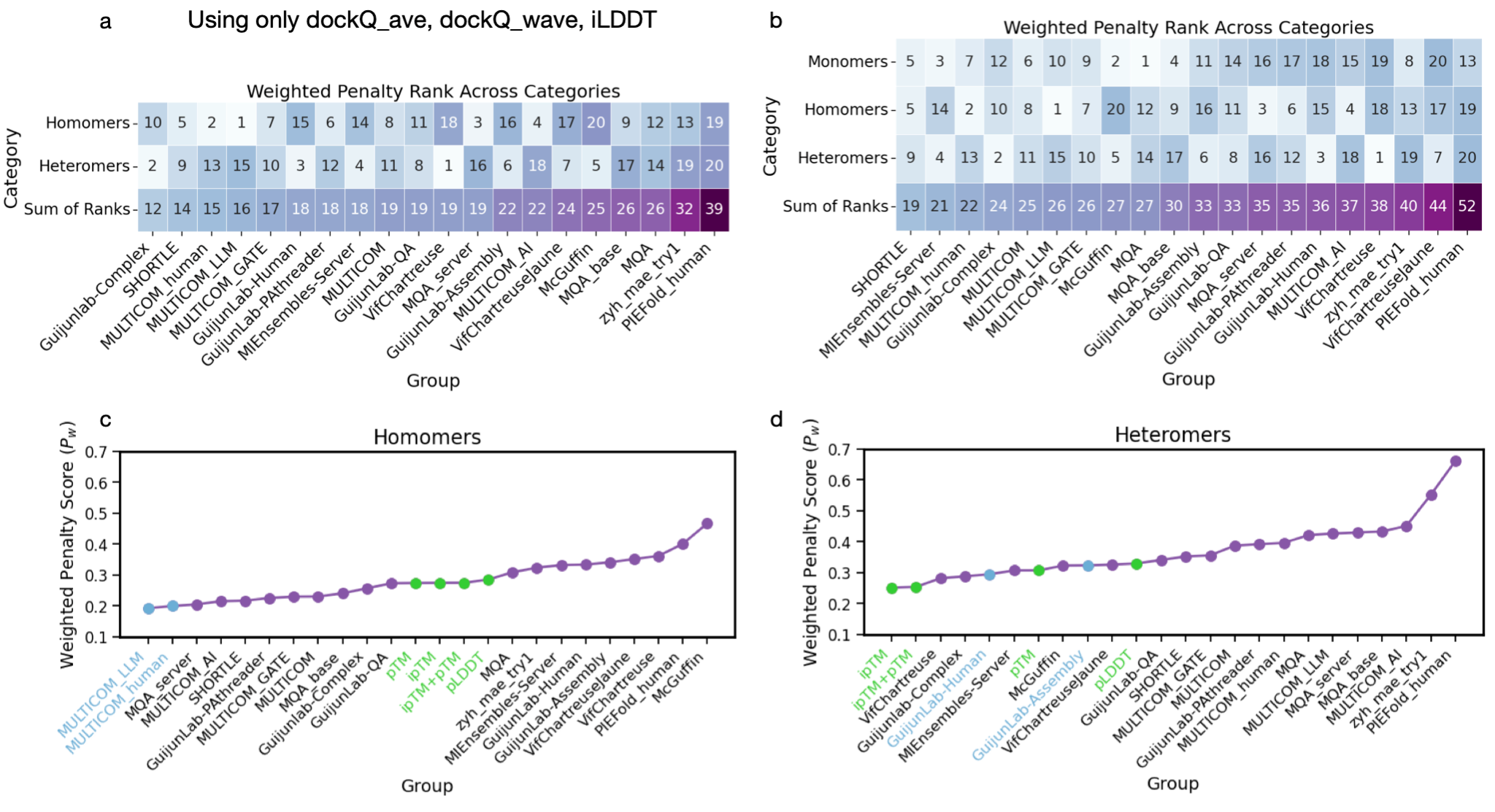


**Figure S3: QMODE3 Weighted Penalty Group Rankings per Target Category Using a Subset of Scores.** The final rank obtained by each predictor group through the QMODE3 covariance-weighted penalty with a subset of oligomer-focused scores is shown for the oligomeric categories (a) and for all three target categories (b). The sum of ranks obtained across each category is also shown for each group. (c-d) These QMODE3 weighted penalty values obtained from each oligomeric category are plotted against ranked predictor groups. Groups that were robustly in the top five positions for variations of the analysis that used all scores (including a traditional Z-score ranking that equally weighted score contributions – see Methods) are highlighted in light blue. Baseline methods (i.e. evaluation of MassiveFold models predicted solely through its internal confidence metrics) are highlighted in green.


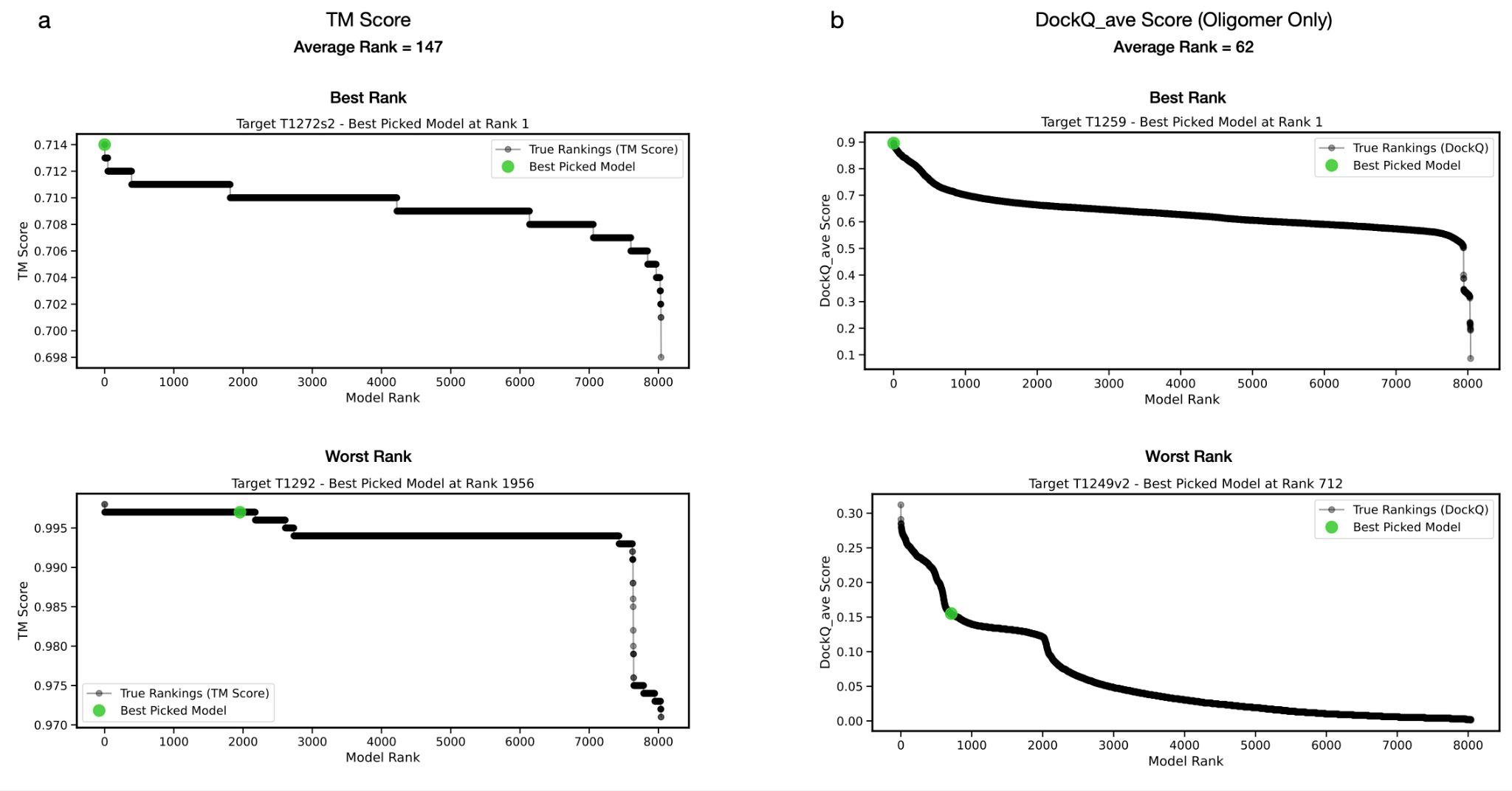


**Figure S4: Best Model Selection in the Context of True Rankings.** For each target, we examined which model (from any predictor group) achieved the lowest ranking error. That model was then ranked in the true model list based on DockQ_ave (for oligomers) or TM score (chosen as a less DockQ-correlated global accuracy metric that is applicable to all target categories). The mean true rank for the best-selected model across all targets was 147 (TM score analysis) or 62 (DockQ_ave analysis). Best and worst examples for each score are shown with their full rankings.
